# Legislation

**Published:** 1898-02

**Authors:** 


### LEGISLATION.

*New York State.* As usual, the Empire State again takes the lead in various directions of great interest to veterinarians.

The most important is Senate Bill No. 89, introduced by Mr. Wray. It has for its object the extension of the time allowed for registration with the Board of Regents, without examination, of all graduates whose diplomas are issued before July 1, 1898. This measure has been introduced at the request of a large body of veterinary students now attending the veterinary colleges in New York City, and as so much feeling and criticism have been engendered by the very high standard raised in New York State, which threatens to close at least two of the schools and to deny the other a sufficient number of students to provide for the future needs of the State in qualified veterinarians, it is thought that this measure will be fought for very hard by those directly interested in what it contends for.

Assembly Bill No. 283, introduced by Mr. Sullivan, is intended to relieve the veterinarians of Greater New York City from jury duty, in keeping with the other scientific professions. This relief should be afforded to all the State or to none, though there is the danger-side to all such legislation. Jury service and its high duties are among best safeguards of our nation, and these places should exempt from service the smallest number of educated men and good citizens it is possible to do, and every man should endeavor to throw around the bench of justice the strongest safeguards possible, lest these important posts fall as prey to the professional juryman, the jury-fixer, and the cause of justice becomes feeble in her own temple.

Assembly Bill No. 300, introduced by Mr. Coughtry, and referred to the Committee on Public Health, is the same measure that has been twice defeated, and is planned to reopen registration of new graduates for a period of two months, to enable a number of unqualified men throughout the State to register. Those interested in this measure claim that they were otherwise qualified to do this under the law that closed the doors in 1895 to these registrations, and, having neglected to take advantage of these provisions, now ask special legislation in their behalf in the form of an enabling act. This measure should be scotched in committee, and should be buried so deep that it will never again be resurrected. Veterinarians all over the State should write and ask for the defeat of this measure.

